# Retention of an endoscopic videocapsule on inflammatory polyposis of the small bowel

**DOI:** 10.1016/j.amsu.2021.102323

**Published:** 2021-04-16

**Authors:** Taoufik El abbassi, Wafaa Hliwa, Yassine El Berni, Salahedine Elmassi, Wafaa Badre, M. Rachid Lefriyekh

**Affiliations:** a Department of General Surgery, Ibn Rochd University Hospital Center, Casablanca, Morocco; b Gastroenterology Department, Ibn Rochd University Hospital Center, Casablanca, Morocco; c Faculty of Medicine and Pharmacy, Hassan II University, Casablanca, Morocco

**Keywords:** Anemia, Digestive haemorrhage, Video capsule endoscopy

## Abstract

The Video Capsule Diagnostic Imaging is a technique for exploring the digestive tract, particularly the small bowel. It is indicated for any unexplained digestive bleeding or as a means of monitoring intestinal polyposis or inflammatory diseases. This videocapsule is not digestible, and the risk of its retention, symptomatic or not, is not negligible following an inflammatory, anastomatous or tumoral stenosis. This retention or blockage is defined by the presence of the Video Capsule in the digestive tract at least two weeks after ingestion. Surgical approach is considered effective to retrieve the retained capsule, treat the pathology responsible and prevent acute complications.

We report the case of retention of a video capsule in a young patient with severe anaemia due to inflammatory polyposis of the small bowl, whose removal required surgery to extract the capsule and resect the segment of the small intestine stenosis by the polyps.

## Highlights

• The video Capsule Endoscopy is the most sensitive examination for the detection of inflammatory lesions of the small bowel.
• It does not currently allow a biopsy but it is indicated for unexplained or occult digestive bleeding.
• Its main limitation is the risk of blockage in a stenosis with or without occlusion.
• Surgical approach is considered effective to retrieve the retained capsule, and treat the pathology responsible.

## 1 Introduction

**Fig. 1:**
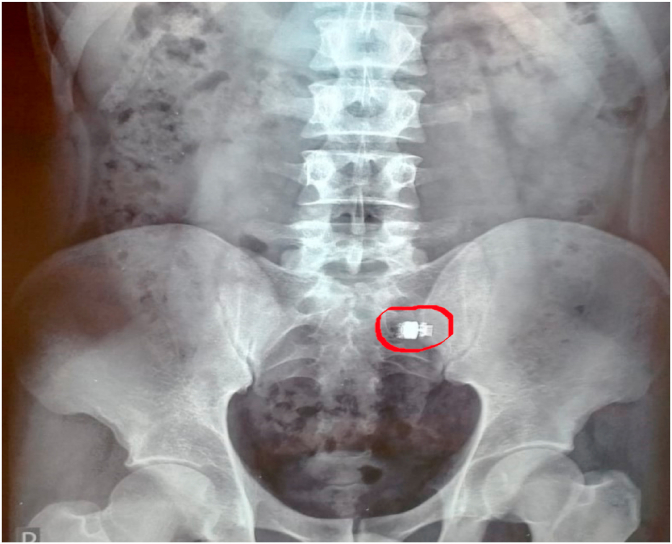
Image of capsule retention on an unprepared abdominal X-ray.

**Fig. 2:**
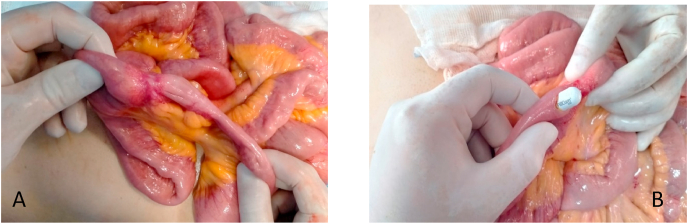
A- Intraoperative image of the bowel stenosis B- Image of capsule extraction.

Video Capsule Endoscopy (VCE) is the most sensitive examination for the detection of inflammatory lesions of the small bowel. Its sensitivity and specificity is close to 90% [1]. Although it does not currently allow a biopsy, it remains an essential non-invasive diagnostic technique indicated for unexplained or occult digestive bleeding manifested by an anemia [2]. Its main limitation is the risk of blockage in a stenosis with or without occlusion in 1–5% [3]. This retention rate varies according to the pathology responsible for the stenosis and the risk is higher in case of known Crohn's disease, but also in case of taking non-steroidal anti-inflammatory drugs, a history of digestive radiotherapy or tumors. (see Fig. 1, Fig. 2)

The purpose of this work is to present a case of retention of a videocapsule at the small bowel treated surgically. the work has been reported in line with the SCARE 2020 criteria [4].

## 2 Observation

The patient is 30 years old, with no particular pathological history, followed for a chronic anemia unexplained for 3 months, without externalized digestive haemorrhage or transit disorder, all evolving in a context of conservation of the general state. Clinical examination found a stable hemodynamic patient, with discolored conjunctivae, slight peri-umbilical abdominal tenderness, without palpable abdominal mass or hepatomegaly or splenomegaly. The rectal examination found no bleeding stigma. On the biological check-up, the hemoglobin level was 7g/dl, GMV 70.5μ3, platelets 350000ele/mm3. His haemostasis assessment was correct. eosogastric fibroscopy and colonoscopy were normal. An enteroscanner had objectified a thickening of only a few parts of the small intestine, hence the indication for an exploration by a videocapsule, which had detected a fern leaf lesion at the jejunum proximal substenosing preventing the progression of the capsule beyond. In front of the absence of elimination of the capsule after 15 days of its digestion, without occlusive syndrome, the retention complication was retained which required a surgical extraction.

Surgical exploration found a capsule blocked at 1 m from the duodeno-jejunal angle by polyps. The extraction of the capsule was done as well as a complete resection of the polypoid lesion on the bowel. The histological study showed inflammatory polyps with complete resection.

The postoperative follow-up was simple, with normalization of hemoglobin levels for a year's decline.

## 3 Discussion

The endoscopic videocapsule has become the reference examination for exploring the lumen of the small intestine. It is mainly indicated for the diagnosis of unexplained digestive bleeding, or in the context of Crohn's disease in the search for mucosal lesions when morphological and endoscopic examinations of the small intestine with biopsies are negative [5,6]. It is also indicated in celiac disease, digestive polyposis, or small bowel carcinoid tumors. Its disadvantage is that it does not allow biopsy or therapeutic procedures to be performed.

The main fearsome complication of endoscopic videocapsule is its retention in case of digestive stenosis with or without occlusion. It is therefore necessary to carefully check the patient's history (surgery, radiotherapy treatment, non-steroidal anti-inflammatory drugs) before proceeding with the placement of the capsule.

The International Conference on Capsule Endoscopy (ICCE) defined Videocapsule retention in 2005 as the presence of a capsule in the gastrointestinal tract for at least two weeks requiring medical, endoscopic or directed surgical intervention [7]. It must be differentiated from stagnation secondary to intestinal transit. The retention rate found in different studies varies according to the pathology or symptoms suggestive of stenosis. It appears to be between 1 and 5% [2,6].

The most frequent causes of this retention are stenosis due to non-steroidal anti-inflammatory drugs, radiation enteritis, intestinal tumors, surgical anastomoses and especially Crohn's disease [8]. Capsule retention is usually asymptomatic but sometimes causes intestinal subocclusion. This retention indicates the presence of an underlying pathology and thus helps to identify the etiology and site of the obstruction [9]. In the absence of symptoms, an X-ray of the abdomen is recommended two weeks after ingestion [10]. Surgery is indicated for acute small bowel obstruction with the benefit of removing the Videocapsule and treating the obstructive lesion. However, in the absence of obstruction, medical management may initially be attempted such as corticosteroid therapy, which may be instituted for Crohn's disease flare-ups and for active narrowing which may allow subsequent passage of the videocapsule. Thus, endoscopic extraction through the enteroscope can be tried [11]. The prevention of retention is based on interrogation, enteroscanner or enteroMRI, and for patients likely to have small bowel stenosis, a capsule containing lactose that disintegrates in the digestive tract (Patency Agile capsule) is proposed [12].

## 4 Conclusion

The endoscopic video-capsule for the small bowel has revolutionized the exploration of the small bowel in the front of unexplained digestive bleeding or suspicion of Crohn's disease, localized exclusively to the small intestine. It represents the reference examination after a normal gastrointestinal endoscopy and colonoscopy. However, retention of the capsule remains one of its main limitations, which sometimes requires surgery to extract it.

## Ethical approval

I declare on my honor that the ethical approval has been exempted by my establishment.

## Sources of funding

None.

## Author contribution

Taoufik El abbassi:Corresponding author writing the paper and operating surgeon.

Wafaa Hliwa writing the paper.

Yassine El Berni writing the paper.

Salahedine Elmassi writing the paper.

Wafaa Badre study concept.

M. Rachid Lefriyekh study concept.

## Trial registry number

It's not a study but a case report that why we don't number of registration.

## Guarantor

Pr Elabbassi taoufik.

## Consent

Written informed consent was obtained from the patient for publication of this case report and accompanying images. A copy of the written consent is available for review by the Editor-in-Chief of this journal on request.

## Contribution of the authors

All authors contributed equally to the conduct of this work. They also declare that they have read and approved the final version of the manuscript.

## Declaration of competing interest

The authors declare having no conflicts of interest for this article.
